# Efficacy and Safety of Electrohydraulic Lithotripsy Using Peroral Cholangioscopy under Endoscopic Retrograde Cholangiopancreatography Guidance in Older Adults: A Single-Center Retrospective Study

**DOI:** 10.3390/medicina59040795

**Published:** 2023-04-19

**Authors:** Koji Takahashi, Hiroshi Ohyama, Yuichi Takiguchi, Yu Sekine, Shodai Toyama, Nana Yamada, Chihei Sugihara, Motoyasu Kan, Mayu Ouchi, Hiroki Nagashima, Yotaro Iino, Yuko Kusakabe, Kohichiroh Okitsu, Izumi Ohno, Naoya Kato

**Affiliations:** 1 Department of Gastroenterology, Graduate School of Medicine, Chiba University, Chiba 260-8670, Japan; 2 Department of Medical Oncology, Graduate School of Medicine, Chiba University, Chiba 260-8670, Japan

**Keywords:** common bile duct stone, elderly, electrohydraulic lithotripsy, endoscopic retrograde cholangiopancreatography, safety

## Abstract

*Background and objectives:* The safety of electrohydraulic lithotripsy (EHL) in older adults remains unclear. We aimed to investigate the efficacy and safety of EHL using peroral cholangioscopy (POCS) under endoscopic retrograde cholangiopancreatography (ERCP) guidance in older adults aged ≥80 years. *Materials and Methods:* This retrospective clinical study was conducted at a single center. Fifty patients with common bile duct stones who underwent EHL using POCS under ERCP guidance at our institution, between April 2017 and September 2022, were enrolled in this study. The eligible patients were divided into an elderly group (*n* = 21, age ≥80 years) and a non-elderly group (*n* = 29, age ≤79 years), and were analyzed. *Results:* A total of 33 and 40 EHL procedures were performed in the elderly and non-elderly groups, respectively. After excluding cases in which stone removal was performed at other institutions, complete removal of common bile duct stones was confirmed in 93.8% and 100% of the elderly and non-elderly groups, respectively (*p* = 0.20). The mean number of ERCPs required for complete removal of bile duct stones was 2.9 and 4.3 in the elderly and non-elderly groups, respectively (*p* = 0.17). In the EHL session, the overall occurrence of adverse events was eight and seven in the elderly (24.2%) and non-elderly (17.5%) groups, respectively; however, the difference was insignificant (*p* = 0.48). *Conclusions:* EHL using POCS under ERCP guidance is effective in patients aged ≥80 years and there was no significant increase in adverse event rates compared to those aged ≤79 years.

## 1. Introduction

Choledocholithiasis is often encountered in daily clinical practice and is a potential risk factor for acute cholangitis, pancreatitis, or obstructive jaundice. The European Society of Gastrointestinal Endoscopy recommends stone removal for patients with common bile duct (CBD) stones, symptomatic or not, who are fit enough to tolerate the intervention [1]. Endoscopic retrograde cholangiopancreatography (ERCP) is the standard procedure used for CBD stone removal. Various techniques have been developed for CBD stone dissolution, including balloon extraction, basket extraction, papillary balloon dilatation, mechanical lithotripsy, and stent insertion [2]. However, CBD stone removal remains challenging in some cases. Difficult CBD stones often require multiple ERCP sessions, which increases the risk of adverse events. Electrohydraulic lithotripsy (EHL) using peroral cholangioscopy (POCS) under ERCP guidance is considered an option for the treatment of difficult CBD stones. EHL is particularly effective in patients with large stones that are difficult to grasp due to breakup using a mechanical lithotripter or in those with bile duct strictures [3]. To date, there have been several reports on the safety of ERCP in the elderly; however, there are few comprehensive reports on the efficacy and safety of EHL using POCS under ERCP guidance in the elderly. To investigate this issue, we retrospectively evaluated the clinical outcomes and procedure-related adverse event rates of EHL using POCS under ERCP guidance in an elderly group (age ≥80 years) and non-elderly group (age ≤79 years) at our institution.

## 2. Patients and Methods

### 2.1. Study Design

This retrospective clinical study was conducted at a single center. Fifty consecutive patients with CBD stones who underwent EHL using POCS under ERCP guidance at our institution, between April 2017 and September 2022, were enrolled in this study. The exclusion criteria were as follows: (1) patients aged <20 years; (2) patients with surgical altered gastrointestinal anatomy, other than the distal gastrectomy Billroth I reconstruction; (3) patients with unclear background and treatment information; and (4) patients judged as inappropriate by the investigators.

The baseline characteristics of eligible patients included age, sex, Eastern Cooperative Oncology Group performance status (ECOG-PS), Charlson comorbidity index, previously attempted methods of stone removal, maximum CBD stone diameter, and presence of multiple stones. Clinical outcomes included the total number of EHL per patient, confirmation of complete removal of the CBD stone, procedure time, and adverse events. Patients were retrospectively examined using their medical records and data from an endoscopic database at our institution. The data were analyzed by patient and EHL session. Eligible patients were then divided into an elderly group (aged ≥80 years) and a non-elderly group (aged ≤79 years) and compared.

### 2.2. Definitions

The size and number of CBD stones were evaluated by cholangiography. The procedure time from duodenoscope insertion to removal was measured. The complete removal of CBD stones was defined as the confirmation of the absence of stones in the bile duct by cholangiography. The definitions and severity of each adverse event were defined according to the lexicon of the American Society for Gastrointestinal Endoscopy [4]. To explain the details, an adverse event was defined as an event that caused a prolongation of the hospital stay, medical consultation, or another procedure. Severity was defined as follows: an event requiring unplanned transfusion, ventilation support, additional endoscopic or radiological intervention, intensive care unit admission, or prolonged hospitalization for 4–10 days, which was defined as moderate. An adverse event which required surgical intervention, an intensive care unit stay of >1 day, or prolonged hospitalization for >10 nights was defined as severe. If an adverse event did not correspond to any of these, it was defined as mild. The ECOG-PS is a score that expresses the patient’s daily living abilities, and the key elements of the ECOG-PS scale first appeared in the medical literature in 1960 [5]. The Charlson comorbidity index, which was proposed in 1987, is an index that evaluates comorbidities that contribute to death and is reported to be correlated with short-term mortality risk [6]. The ECOG-PS and Charlson comorbidity index were calculated from the patient’s status records immediately prior to their first EHL.

### 2.3. Techniques

**Figure 1:**
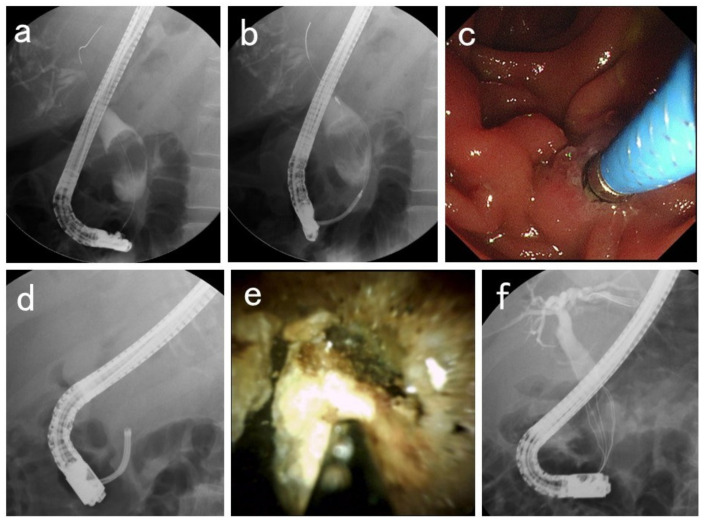
Electrohydraulic lithotripsy techniques using peroral cholangioscopy under endoscopic retrograde cholangiopancreatography guidance. (**a**) Cholangiography image showing a large common bile duct stone. (**b**) Stone removal is impossible using standard techniques. (**c**,**d**) A cholangioscope is inserted into the bile duct. (**e**) Electrohydraulic lithotripsy was performed under cholangioscopic guidance using an electrohydraulic lithotripsy probe. (**f**) Stone removal was performed using the standard technique after fragmentation of the common bile duct stone.

For patients with acute cholangitis, EHL was not performed in the state of cholangitis, but was performed after it subsided by stent placement or nasobiliary drainage. All patients fasted from the morning of the day of the treatment. EHL using POCS under ERCP guidance was performed as follows: prophylactic intravenous antibiotics were administered prior to the ERCP. Carbon dioxide insufflation was used during the procedure unless contraindicated. All patients underwent conscious sedation with the administration of a combination of midazolam and pethidine hydrochloride, or fentanyl and propofol. An oblique-viewing duodenoscope (TJF-260V, TJF-Q290V, Olympus Corporation, Tokyo, Japan) was inserted orally to reach the duodenal papilla, and an ERCP catheter (PR-104Q-1, Olympus, Tokyo, Japan; MTW ERCP catheter, ABIS, Hyogo, Japan) was inserted into the bile duct followed by cholangiography. After imaging the bile duct with a contrast medium, a 0.025-inch guidewire (VisiGlide2, Olympus Corporation, Tokyo, Japan; M-Through, ASAHI INTECC, Aichi, Japan; EndoSelector; Boston Scientific, Marlborough, MA, USA) was placed in the bile duct. Then, a cholangioscope (CHF-B260, CHF-B290, Olympus Corporation, Tokyo, Japan; SpyScope DS, SpyScope DS II; Boston Scientific, Marlborough, MA, USA) was inserted into the bile duct. An electrohydraulic shock wave generator was used to generate shock waves. EHL was performed under POCS guidance using a 1.9 French gauge EHL probe. After fragmentation, CBD stone removal was performed using ERCP techniques, such as a basket or balloon extraction (Figure 1). After treatment, the patient rested in bed for 2 h, and a blood test was performed the following day. Imaging tests, such as ultrasonography and computed tomography, were promptly performed if procedure-related adverse events were suspected.

### 2.4. Statistical Analysis

The data are presented as means with standard deviations or numbers with percentages. Pearson’s chi-squared test was used to assess the categorical data, whereas the Mann–Whitney U test was used to assess the quantitative data. Statistical significance was set at *p* < 0.05. All statistical analyses were performed using the bell curve for Excel (Social Survey Research Information Co., Ltd., Tokyo, Japan).

## 3. Results

Fifty eligible patients (28 men and 22 women) were included in this study. The age range of the patients was 36–94 years, with a mean age of 75.1 years. All eligible patients had previously attempted non-EHL stone removal, but it was unsuccessful. Endoscopic sphincterotomy (EST) had been performed previously on all eligible patients. Table 1 shows the baseline characteristics of all the eligible patients. The mean ECOG-PS was 1.0. The mean Charlson comorbidity index was 1.3. As previously attempted methods of stone removal, removal by a basket catheter, balloon catheter, or both was performed in all cases. Endoscopic papillary balloon dilatation was performed in 16.0% of patients, and an endoscopic mechanical lithotripsy was attempted in 56.0% of patients. The mean maximum diameter of the bile duct stones was 15.0 mm. The proportion of patients with multiple stones was 70.0%. The mean number of EHL was 1.4 times. In 42 patients, excluding 8 who underwent stone removal at another hospital after EHL, 41 patients (97.6%) had complete CBD stone removal. The mean number of ERCP procedures performed prior to complete stone removal was 3.5 times.

The procedure times and adverse events per EHL session are shown in Table 2. In total, 73 EHL sessions were conducted. The mean procedure time was 57.7 min. Adverse events occurred in 20.5% of all EHL procedures. Cholangitis occurred in 12 patients (16.4%), and its severity was mild in all patients. Pancreatitis occurred in two patients (2.7%) with mild severity. Peritonitis occurred in one patient (1.4%) with moderate severity. Pneumonia occurred in one patient (1.4%) with mild severity. No procedure-related deaths or sedation-related problems were reported.

All eligible patients were divided into an elderly group (age ≥80 years) and a non-elderly group (age ≤79 years). Table 3 shows the results of the comparison of baseline characteristics and final treatment results of the elderly and non-elderly groups. There were 21 and 29 patients in the elderly and non-elderly groups, respectively. The mean age was 84.6 and 68.2 years in the elderly and the non-elderly groups, respectively (*p* < 0.001). The proportion of males was 57.1% and 55.2% in the elderly and non-elderly groups, respectively (*p* = 0.89). The mean ECOG-PS was 1.7 and 1.0 in the elderly and non-elderly groups, respectively (*p* < 0.001). The mean Charlson comorbidity index was 1.5 and 1.3 in the elderly and non-elderly groups, respectively (*p* = 0.17). Regarding previously attempted methods of stone removal, other than basket catheters and balloon catheters, the proportion of attempted endoscopic papillary balloon dilatation was 23.8% and 10.3% in the elderly and non-elderly groups, respectively (*p* = 0.20). The proportion of attempted endoscopic mechanical lithotripsy was 47.6% and 62.1% in the elderly and non-elderly groups, respectively (*p* = 0.31). The mean maximum diameter of the CBD stones was 16.1 mm and 14.1 mm in the elderly and non-elderly groups, respectively (*p* = 0.085). The percentage of patients with multiple CBD stones was 61.9% and 75.9% in the elderly and non-elderly groups, respectively (*p* = 0.29). A total of 33 and 40 EHL procedures were performed in the elderly and non-elderly groups, respectively. The proportion of patients who underwent stone removal at another hospital after EHL was 23.8% and 10.3% in the elderly and non-elderly groups, respectively (*p* = 0.35). Complete removal of CBD stones was confirmed in 15 of 16 cases (93.8%) and in all 26 cases (100%) in the elderly and non-elderly groups, respectively (*p* = 0.20). The mean number of ERCPs required for complete removal of bile duct stones was 2.9 and 4.3 in the elderly and non-elderly groups, respectively (*p* = 0.17).

The clinical outcomes for each EHL session are shown in Table 4. The mean procedure time was 54.4 and 60.4 min in the elderly and non-elderly groups, respectively (*p* = 0.15). The overall occurrence of adverse events was eight and seven in the elderly (24.2%) and non-elderly (17.5%) groups, respectively, with no significant difference (*p* = 0.48). Cholangitis occurred in both the elderly and non-elderly groups. Pancreatitis occurred only in the non-elderly group, whereas peritonitis and pneumonia occurred only in the elderly group. However, these differences were not statistically significant between the two groups.

## 4. Discussion

The present study investigated the efficacy and safety of EHL using POCS under ERCP guidance in patients aged ≥80 years. Even in patients in whom CBD stone removal was impossible using ERCP without EHL, 97.6% of the CBD stones were completely removed, finally. The adverse event rates were relatively high (20.5%), with mild cholangitis being the most common adverse event. There were no significant differences in the adverse event rates between patients aged >80 years and those aged ≤79 years. Furthermore, no procedure-related deaths occurred.

In EHL, a cholangioscope is inserted into the bile duct using ERCP and then, a probe for EHL is inserted into the bile duct to generate shock waves to crush the CBD stones. Because EHL is relatively labor intensive and costly, it is typically performed only when stone removal is difficult using other techniques. If the distal bile duct diameter is larger than that of the CBD stone, it can be removed with endoscopic papillary balloon dilatation, and if the CBD stone can be grasped with a mechanical lithotripter, it can be crushed and removed. EHL is indicated for CBD stones that are difficult to remove using endoscopic papillary balloon dilatation or endoscopic mechanical lithotripsy. In this study, EST was performed in all eligible patients, and endoscopic papillary balloon dilatation was performed in a small proportion of patients (16.0%); however, endoscopic mechanical lithotripsy was attempted in more than half of the patients. The contents of the endoscopic treatment prior to EHL depended on the environment of each facility where ERCP was performed. Some facilities do not have the devices for endoscopic papillary balloon dilatation or endoscopic mechanical lithotripsy. At our institution, EHL may be performed without endoscopic papillary balloon dilatation or endoscopic mechanical lithotripsy in cases where the stone is larger than the diameter of the distal bile duct or it is expected to be difficult to grasp with a mechanical lithotripter.

In this study, the overall EHL-related adverse event rate was 20.5%, which was higher than that of the conventional ERCP-related procedure. Among the adverse events, cholangitis was the most common at 16.4%. Cholangitis is presumed to be caused by increasing intraductal pressure of the bile duct due to washing the bile duct by injecting saline during the EHL. In this study, all cases of EHL-related cholangitis were mild in severity and improved rapidly without additional intervention. The pancreatitis was presumed to be caused by temporary edema of the duodenal papilla, due to the stimulation by the insertion of the cholangioscope and improved rapidly after the procedure without additional treatment. Peritonitis occurred in one case. Although the cause was not clear, EHL was performed within a short period after cholecystectomy in this case, and it was speculated that this occurred because the severed part of the cystic duct was partially released due to the increasing intraductal pressure of the bile duct. Pneumonia occurred in one case and was considered to be caused by saliva or gastric juice entering the airway during EHL.

Several reports have shown the efficacy of EHL using POCS under ERCP guidance for the removal of CBD stones. Conventionally, reusable cholangioscopes were used for EHL. Single-use cholangioscopes have recently been developed. A reusable cholangioscope can be used repeatedly; therefore, it is cost-effective if it is not broken down. However, even in facilities that have a reusable cholangioscope, most facilities have only one, and it is necessary to interrupt or stop treatment if it breaks down. The disadvantage of single-use cholangioscopes is their high cost. However, the risk of infection through the scope is very low. In 2018, Kamiyama reported that the complete CBD stone removal rate was 98%, with an adverse event rate of 12% for cholangitis and 2.4% for pancreatitis in EHL using single-use cholangioscopes. [3]. In 2020, Murabayashi compared the clinical outcomes of reusable cholangioscopy and single-use cholangioscopes in EHL and found no significant difference in the rate of final stone removal and adverse events between both groups; however, the procedure time was significantly shorter in the single-use cholangioscopy group, and the mean number of endoscopic sessions was significantly lower in the single-use cholangioscopy group [7].

Although there are few reports on the safety of EHL using POCS under ERCP guidance in older adults, there are several reports on the safety of ERCP in older adults. In 2015, a retrospective study showed that ERCP could be safely performed even in those aged ≥80 years, however the sedation-related adverse events increased in a retrospective analysis of 758 patients who underwent ERCP [8]. In 2018, a retrospective study comparing patients aged ≥80 years to those aged ≤65 years who underwent therapeutic ERCP, showed that the rate of difficult cannulation was higher in the ≥80 years group, the mean procedure time was longer in the aged ≥80 years group, and second ERCPs were performed more frequently in the aged ≥80 years group. The overall success and adverse event rates were not significantly different between the two groups [9]. In 2018, a retrospective study comparing patients aged ≥85 years to those aged <85 years who underwent therapeutic ERCP for CBD stones showed no difference in the recurrence rate of stones, adverse event and mortality rates, and the length and cost of hospitalization between the two groups [10]. In older adults, attention should be paid to post-procedural pneumonia. In 2023, a retrospective study showed that patients aged ≥90 years who underwent ERCP for CBD stone removal had a higher incidence of post-ERCP pneumonia than those aged 65–89 years [11]. In addition, there is a report that patients aged ≥90 years require particular attention among the elderly. In 2018, a retrospective study of 137 patients aged ≥85 years who underwent therapeutic ERCP showed that the incidence of adverse events significantly increased in those aged ≥90 years [12].

It is also characteristic of the elderly that the ECOG-PS is often lower than that of the non-elderly. There are several reports on the effect of ECOG-PS on ERCP. In 2019, a retrospective study showed that comparing 287 patients who underwent therapeutic ERCP divided into ECOG-PS 3–4 group and ECOG-PS 0–2 group, the overall adverse events did not significantly differ; however, aspiration pneumonia and heart failure were more likely to occur among patients with ECOG-PS 3–4 [13]. In a retrospective study reported in 2021, 1845 patients who underwent ERCP were divided into two groups: ECOG-PS 0–3 patients and ECOG-PS 4 patients. The pulmonary adverse event rate and severe adverse event rate were significantly higher in the ECOG-PS 4 group [14]. In a retrospective study reported in 2022, 1343 native papillae who underwent therapeutic ERCP for CBDS were divided into two groups: ECOG-PS 0–2 and those with ECOG-PS 3–4. No significant difference was observed between the therapeutic success rates and the overall ERCP-related adverse event rates; however, adverse events were significantly more severe in the ECOG-PS 3–4 group than in the ECOG-PS 0–2 group [15].

In our study, although there was no significant difference in the Charlson comorbidity index between the two groups, the ECOG-PS was significantly lower in the elderly group. In addition, although there was no significant difference, the elderly group tended to have multiple stones. However, there was no significant difference in clinical outcomes, including the occurrence rate of adverse events, between the two groups, and there were no treatment-related deaths. No duodenal perforation or bleeding occurred in this study. EHL does not require a large sphincterotomy because the CBD stones are crushed in the bile duct before stone removal. In addition, EHL does not require excessive dilation of the duodenal papilla, so it is a procedure with relatively little irritation to the duodenal papilla. Therefore, even if mild cholangitis occurs relatively frequently, serious adverse events, such as hemorrhage, perforation, and pancreatitis, may occur less frequently. Currently, EHL is not the first choice for difficult stones because it is costlier and more complicated to prepare than EML or EPBD. If it is resolved, depending on the case, EHL can be the first choice for CBD stones that are difficult to remove with basket or balloon extraction.

In this study, CBD stones, which were difficult to remove using other methods, were completely removed using EHL at a very high rate. The mean procedure time for EHL was 57.7 min, and the occurrence rates of post-procedure cholangitis were 16.4%. Compared to other ERCP procedures, EHL has a relatively long procedure time and a relatively high adverse event rate. However, EHL enables complete removal of difficult stones at a high rate and can be said to be a very useful procedure. There was no significant difference in the clinical outcomes and occurrence rates of adverse events in EHL between patients aged ≥80 years and those aged ≤79 years. EHL can be safely performed in individuals aged >80 years. However, attention should be given to the onset of post-procedural pneumonia.

This study has several limitations. First, it is a retrospective study. Second, the sample size of this study is small. Third, there are case-to-case differences in the endoscopic treatment before EHL. Fourth, there is a difference in the background characteristics between the elderly group and the non-elderly group because propensity score matching was not performed. Finally, the long-term outcomes, such as the recurrence rate of CBD stones after EHL treatment, are unclear.

## 5. Conclusions

In our study, we found that EHL using POCS under ERCP guidance enabled the efficient removal of CBD stones in the elderly group aged ≥80 years, and there was no significant increase in adverse event rates compared with those aged ≤79 years. Our analysis suggests that EHL using POCS under ERCP guidance can be performed equally effectively and safely in the elderly and non-elderly.

## Figures and Tables

**Table 1:** Baseline characteristics of all eligible patients.

Variable	*n* = 50
Age, year, mean (SD)	75.1 (11.5)
Sex, male, *n* (%)	28 (56)
ECOG-PS, mean (SD)	1.0 (1.1)
Charlson comorbidity index, mean (SD)	1.3 (1.1)
Previously attempted methods for stone removal, *n* (%)	
Endoscopic papillary balloon dilatation	8 (16.0)
Endoscopic mechanical lithotripsy	28 (56.0)
Maximum diameter of bile duct stones, mm, mean (SD)	15.0 (4.8)
Multiple stones, *n* (%)	35 (70.0)
Total number of EHL per patient, mean (SD)	1.4 (1.0)
Number of cases in which complete stone removal was finally confirmed, *n* (%)	41/42 (97.6)
Number of ERCPs before complete stone removal, mean (SD)	3.5 (2.4)

SD: standard deviation; ECOG-PS: Eastern Cooperative Oncology Group performance status; EHL: electrohydraulic lithotripsy; ERCP: endoscopic retrograde cholangiopancreatography.

**Table 2:** The procedure time and adverse events per electrohydraulic lithotripsy session.

Variable	*n* = 73
Procedure time, minutes, mean (SD)	57.7 (17.0)
Adverse events, *n* (%)	15 (20.5)
Cholangitis	12 (16.4)
mild	12 (16.4)
Pancreatitis	2 (2.7)
mild	2 (2.7)
Peritonitis	1 (1.4)
moderate	1 (1.4)
Pneumonia	1 (1.4)
mild	1 (1.4)

SD: standard deviation.

**Table 3:** Comparison of baseline characteristics and final treatment results between the elderly and non-elderly groups.

Variable	Elderly	Non-Elderly	*p*-Value
	*n* = 21	*n* = 29	
Age, year, mean (SD)	84.6 (4.2)	68.2 (10.1)	<0.001
Sex, male, *n* (%)	12 (57.1)	16 (55.2)	0.89
ECOG-PS, mean (SD)	1.7 (1.0)	1.0 (1.0)	<0.001
Charlson comorbidity index, mean (SD)	1.5 (1.0)	1.3 (1.0)	0.17
Previously attempted methods for stone removal, *n* (%)			
Endoscopic papillary balloon dilatation	5 (23.8)	3 (10.3)	0.20
Endoscopic mechanical lithotripsy	10 (47.6)	18 (62.1)	0.31
Maximum diameter of bile duct stones, mm, mean (SD)	16.1 (4.3)	14.1 (5.0)	0.085
Multiple stones, *n* (%)	13 (61.9)	22 (75.9)	0.29
Total number of performing EHL	33	40	
Total number of performing EHL per patients, mean (SD)	1.3 (0.6)	1.5 (1.2)	0.86
Number of patients who underwent stone removal at another hospital after EHL, *n* (%)	5 (23.8)	3 (10.3)	0.35
Number of cases in which complete stone removal was finally confirmed, *n* (%)	15/16 (93.8)	26/26 (100)	0.20
Number of ERCPs before complete stone removal, mean (SD)	2.9 (1.4)	4.3 (2.9)	0.17

SD: standard deviation; ECOG-PS: Eastern Cooperative Oncology Group performance status; EHL: electrohydraulic lithotripsy; ERCP: endoscopic retrograde cholangiopancreatography.

**Table 4:** Clinical outcomes per electrohydraulic lithotripsy session.

Variable	Elderly	Non-Elderly	*p*-Value
	*n* = 33	*n* = 40	
Procedure time, minutes, mean (SD)	54.4 (15.7)	60.4 (17.6)	0.15
Adverse events, *n* (%)	8 (24.2)	7 (17.5)	0.48
Cholangitis	6 (18.2)	6 (15.0)	0.72
mild	6 (18.2)	6 (15.0)	0.72
Pancreatitis	0	2 (5.0)	0.19
mild	0	2 (5.0)	0.19
Peritonitis	1 (3.0)	0	0.27
moderate	1 (3.0)	0	0.27
Pneumonia	1 (3.0)	0	0.27
mild	1 (3.0)	0	0.27

SD: standard deviation.

## Data Availability

The datasets generated during and/or analyzed during the current study are not publicly available but are available from the corresponding author upon reasonable request.

## References

[1] Manes G., Paspatis G., Aabakken L., Anderloni A., Arvanitakis M., Ah-Soune P., Barthet M., Domagk D., Dumonceau J.M., Gigot J.F. (2019). Endoscopic management of common bile duct stones: European Society of Gastrointestinal Endoscopy (ESGE) guideline. Endoscopy.

[2] Kedia P., Tarnasky P.R. (2019). Endoscopic Management of Complex Biliary Stone Disease. Gastrointest. Endosc. Clin..

[3] Kamiyama R., Ogura T., Okuda A., Miyano A., Nishioka N., Imanishi M., Takagi W., Higuchi K. (2018). Electrohydraulic Lithotripsy for Difficult Bile Duct Stones under Endoscopic Retrograde Cholangiopancreatography and Peroral Transluminal Cholangioscopy Guidance. Gut Liver.

[4] Cotton P.B., Eisen G.M., Aabakken L., Baron T.H., Hutter M.M., Jacobson B.C., Mergener K., Nemcek A., Petersen B.T., Petrini J.L. (2010). A lexicon for endoscopic adverse events: Report of an ASGE workshop. Gastrointest. Endosc..

[5] Oken M.M., Creech R.H., Tormey D.C., Horton J., Davis T.E., McFadden E.T., Carbone P.P. (1982). Toxicity and response criteria of the Eastern Cooperative Oncology Group. Am. J. Clin. Oncol..

[6] Charlson M.E., Pompei P., Ales K.L., MacKenzie C.R. (1987). A new method of classifying prognostic comorbidity in longitudinal studies: Development and validation. J. Chronic. Dis..

[7] Murabayashi T., Ogawa T., Koshita S., Kanno Y., Kusunose H., Sakai T., Masu K., Yonamine K., Miyamoto K., Kozakai F. (2020). Peroral Cholangioscopy-guided Electrohydraulic Lithotripsy with a SpyGlass DS Versus a Conventional Digital Cholangioscope for Difficult Bile Duct Stones. Intern. Med..

[8] Finkelmeier F., Tal A., Ajouaou M., Filmann N., Zeuzem S., Waidmann O., Albert J. (2015). ERCP in elderly patients: Increased risk of sedation adverse events but low frequency of post-ERCP pancreatitis. Gastrointest. Endosc..

[9] Yang J.H., Li W., Si X.K., Zhang J.X., Cao Y.J. (2018). Efficacy and Safety of Therapeutic ERCP in the Elderly: A Single Center Experience. Surg. Laparosc. Endosc. Percutan. Tech..

[10] Iida T., Kaneto H., Wagatsuma K., Sasaki H., Naganawa Y., Nakagaki S., Satoh S., Shimizu H., Nakase H. (2018). Efficacy and safety of endoscopic procedures for common bile duct stones in patients aged 85 years or older: A retrospective study. PLoS ONE.

[11] Jalal M., Khan A., Ijaz S., Gariballa M., El-Sherif Y., Al-Joudeh A. (2023). Endoscopic removal of common bile duct stones in nonagenarians: A tertiary centre experience. Clin. Endosc..

[12] Takahashi K., Tsuyuguchi T., Sugiyama H., Kumagai J., Nakamura M., Iino Y., Shingyoji A., Yamato M., Ohyama H., Kusakabe Y. (2018). Risk factors of adverse events in endoscopic retrograde cholangiopancreatography for patients aged ≥85 years. Geriatr. Gerontol. Int..

[13] Takahashi K., Nihei T., Aoki Y., Nakagawa M., Konno N., Munakata A., Okawara K., Kashimura H. (2019). Efficacy and safety of therapeutic endoscopic retrograde cholangiopancreatography in patients with native papillae with a performance status score of 3 or 4: A single-center retrospective study. J. Rural Med..

[14] Kitano R., Inoue T., Ibusuki M., Kobayashi Y., Ohashi T., Sumida Y., Nakade Y., Ito K., Yoneda M. (2021). Safety and Efficacy of Endoscopic Retrograde Cholangiopancreatography in Patients with Performance Status 4. Dig. Dis. Sci..

[15] Saito H., Kadono Y., Shono T., Kamikawa K., Urata A., Nasu J., Imamura H., Matsushita I., Kakuma T., Tada S. (2022). Endoscopic retrograde cholangiopancreatography for bile duct stones in patients with a performance status score of 3 or 4. World J. Gastrointest. Endosc..

